# On flow fluctuations in ruptured and unruptured intracranial aneurysms: resolved numerical study

**DOI:** 10.1038/s41598-024-70340-7

**Published:** 2024-08-23

**Authors:** Feng Huang, Gábor Janiga, Philipp Berg, Seyed Ali Hosseini

**Affiliations:** 1 Laboratory of Fluid Dynamics and Technical Flows, Otto-von-Guericke-University Magdeburg, D-39106 Magdeburg, Germany; 2 Department of Mechanical and Process Engineering, ETH Zürich, 8092 Zürich, Switzerland; 3 Research Campus STIMULATE, Otto-von-Guericke-University Magdeburg, D-39106 Magdeburg, Germany; 4 Department of Medical Engineering, Otto-von-Guericke-University Magdeburg, D-39106 Magdeburg, Germany

**Keywords:** Biomedical engineering, Fluid dynamics

## Abstract

Flow fluctuations have emerged as a promising hemodynamic metric for understanding of hemodynamics in intracranial aneurysms. Several investigations have reported flow instabilities using numerical tools. In this study, the occurrence of flow fluctuations is investigated using either Newtonian or non-Newtonian fluid models in five patient-specific intracranial aneurysms using high-resolution lattice Boltzmann simulation methods. Flow instabilities are quantified by computing power spectral density, proper orthogonal decomposition, and fluctuating kinetic energy of velocity fluctuations. Our simulations reveal substantial flow instabilities in two of the ruptured aneurysms, where the pulsatile inflow through the neck leads to hydrodynamic instability, particularly around the rupture position, throughout the entire cardiac cycle. In other monitoring points, the flow instability is primarily observed during the deceleration phase; typically, the fluctuations begin just after peak systole, gradually decay, and the flow returns to its original, laminar pulsatile state during diastole. Additionally, we assess the rheological impact on flow dynamics. The disparity between Newtonian and non-Newtonian outcomes remains minimal in unruptured aneurysms, with less than a 5% difference in key metrics. However, in ruptured cases, adopting a non-Newtonian model yields a substantial increase in the fluctuations within the aneurysm sac, with up to a 30% higher fluctuating kinetic energy compared to the Newtonian model. The study highlights the importance of using appropriate high-resolution simulations and non-Newtonian models to capture flow fluctuation characteristics that may be critical for assessing aneurysm rupture risk.

## Introduction

Intracranial aneurysms are local dilatations in cerebral arteries, highly prevalent in adults ($\sim$3.3%) ^1,2^. With the development of non-invasive medical imaging techniques, unruptured intracranial aneurysms are being increasingly detected. While some patients may remain unaffected throughout their life, the risk of rupture stays present, which can lead to severe disabilities ^3,4^. Most treatments involve surgical or minimally-invasive endovascular interventions, which may result in the risk of rupture. To address this, a number of attempts have been made to systematically quantify rupture risk. E.g., Greving et al. ^5^ developed a risk score system (PHASES) used to predict the risk of aneurysm rupture based on various patients and aneurysm characteristics, such as size and location of aneurysms, etc. Despite lower risk scores indicating that there is no need for further treatment, such aneurysms may still rupture, pointing to the need for further research to better understand and predict the rupture risk and improve patient treatment outcomes.

It has been argued that hemodynamics play an important role in the rupture of intracranial aneurysms ^6^. The presence of complex flow patterns within the aneurysm sac has been hypothesized to be a potential contributor to the rupture of aneurysms ^7,8^. Many numerical studies observing non-laminar blood flow behavior associated with intracranial aneurysms were documented ^9–12^. Kurokawa et al. ^13^ reported sound at frequencies in the range of 150-800 Hz caused by flow-induced vibration of the aneurysm wall. Steiger et al. ^14^ reported low-frequency flow fluctuations in cerebral saccular aneurysms of six patients out of twelve using intraoperative Doppler recording. A spectral analysis and concomitant glass model studies showed a correlation between flow fluctuation and aneurysm type. Unexpected vibrations were also reported in experimental aneurysm studies, which are possibly associated with fluctuating flows  ^15–19^. The significance of fluctuations in blood flow has been thoroughly explored and discussed by Steinman et al. ^20^. They found in the study ^21^ that significant narrow-band vibrations ranging from 100 to 500 Hz could be detected in two out of the three aneurysms. Furthermore, the pulsatile nature of blood flow has been observed to influence aneurysm stability in both numerical and experimental studies ^22–24^. It is indeed known that a pulsatile flow can become transitional in a straight pipe during the deceleration phase of the oscillation at Reynolds numbers below 2000  ^25^. This explains why several studies discovered indications of transitional flow in cerebral vessels, despite the low Reynolds numbers found there, and suggesting an elevated risk of rupture ^22,26^.

Computational fluid dynamics (CFD), due to its non-invasive nature, is increasingly used to investigate the hemodynamics in aneurysms. Numerous CFD studies have been conducted to study flow fluctuations in aneurysms ^6,27–30^. Baek et al. ^31^ reported flow instabilities occurring during the deceleration phase of a cardiac cycle at the internal carotid artery and concluded that the occurrence of low-frequency fluctuations depended on the geometry. Many researchers  ^26,32–34^ confirmed the presence of high-frequency fluctuations in patient-specific intracranial aneurysms, including both terminal and lateral cases. Still, many CFD studies may be overlooking high-frequency flow instabilities due to inappropriate solver properties and/or inadequate resolutions and boundary conditions  ^35,36^. Since a generalizable criterion for assessing the risk of rupture remains elusive ^37^, we attempt to contribute to the discussion on this topic via lattice Boltzmann method (LBM) simulation of a pool of patient-specific configurations with known outcome.

## Methods

It is confirming that all methods were carried out in accordance with relevant guidelines and regulations. Besides, all experimental protocols were approved by the University Hospital Magdeburg and it is confirmed that informed consent was obtained from all subjects and/or their legal guardian(s).

### Case description

**Figure 1:**
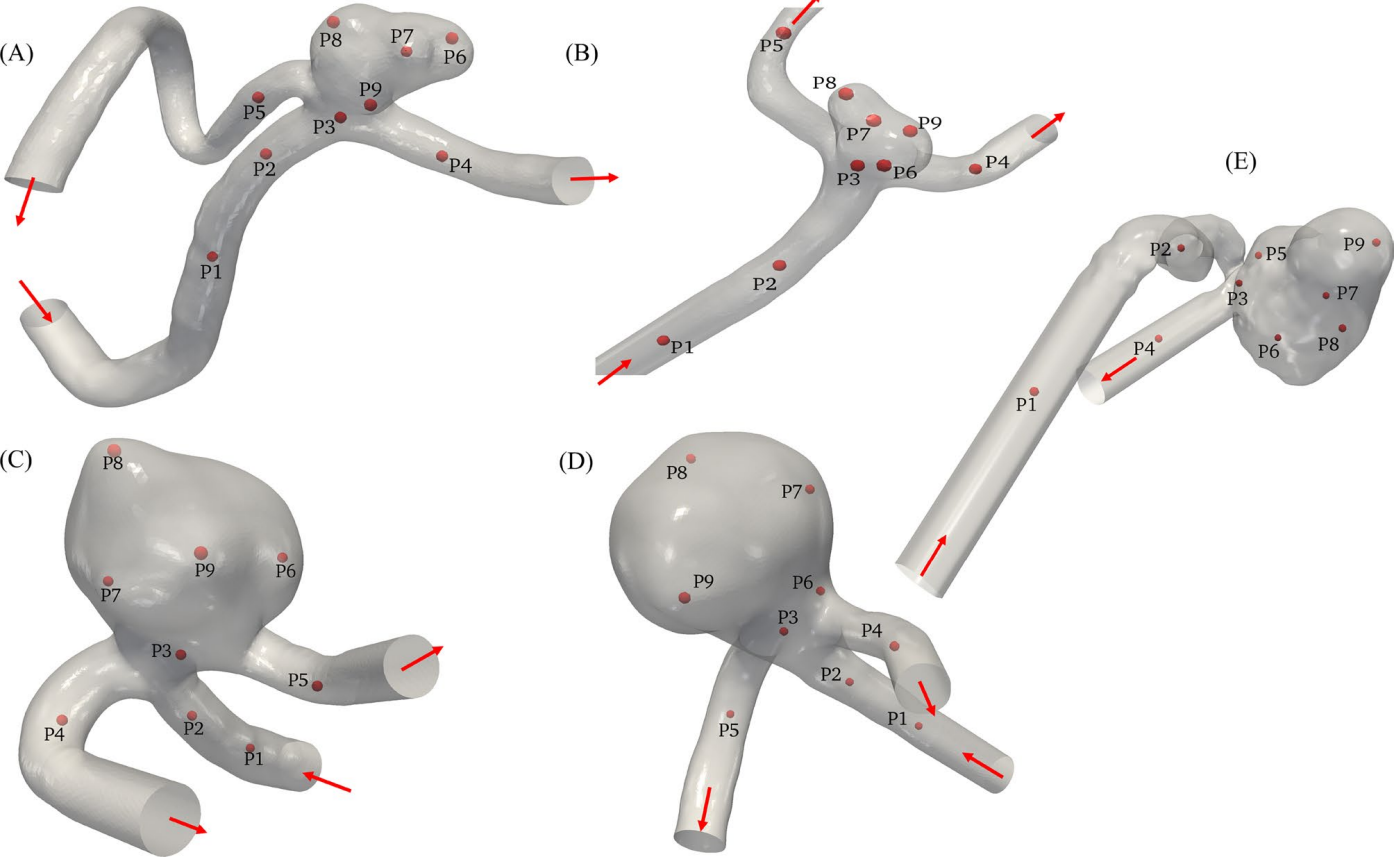
Geometries of the five patient-specific aneurysms (Case A, B, C, D, E) employed in simulations. The red arrows represent the blood flow direction. Note that point P6 corresponds to the ruptured location in case A, while the ruptured points are unknown in other patients.

This study is carried out considering five patient-specific intracranial aneurysms (three ruptured and two unruptured) obtained from previously published research studies ^12,38,39^. The configurations are shown in Figure 1.

The flow values at up to nine points were tracked upstream and downstream of the aneurysms, as well as within the aneurysm sac, in order to monitor any manifestation of possible flow fluctuations. For detailed data, please refer to Table S1. Each case has undergone a thorough investigation to uncover unique characteristics, including known rupture sites, proximal stenosis, and other challenging data sets. For detailed information on imaging, segmentation, and patient history, refer to the supplementary document S. 1.1.

### Numerical method

LBM is an alternative to classical approaches to solve the Navier-Stokes equations. Instead of directly solving the classical mass and momentum balance equations, a system of coupled hyperbolic equations representing the balance of discrete distribution functions, corresponding to a low order truncated version of the Boltzmann equation is solved. This system of equations is readily shown to recover the Navier-Stokes equations in the hydrodynamic limit. The final form of the discrete space/time-evolution equation is:

1 $$\begin{aligned} f_i(\varvec{r}+\varvec{c}_i\delta _t, t+\delta _t) - f_i(\varvec{r}, t) = \frac{\delta _t}{\bar{\tau }}\left( f_i^\text{eq}(\rho ,\varvec{u}) - f_i(\varvec{r}, t)\right) , \end{aligned}$$

where $f_i$ are discrete distribution functions, $\varvec{c}_i$ corresponding discrete velocities, $\bar{\tau }$ is the relaxation time determining the fluid viscosity, $\delta _t$ the time-step size, and $f_i^\text{eq}$ the equilibrium distribution function. For interested readers, detailed review and discussions on the LBM can be found in ^40–42^.

In the present contribution, due to limited stability and operation range of the single relaxation time model ^43,44^, we will use a collision operator that was presented in earlier work and validated for medical flows: A modified version of a central Hermite-based multiple relaxation time operator allowing for independent control of the dissipation rate of normal modes. This collision model has been shown to dramatically improve the stability domain of the solver ^45^. The collision process is carried out in the space of Hermite central moments:

2 $$\begin{aligned} f_i(\varvec{r}+\varvec{c}_i\delta _t, t+\delta _t) - f_i(\varvec{r}, t) = \varvec{\mathscr {T}}^{-1}\varvec{S} \varvec{\mathscr {T}} \left( \varvec{f}^\text{eq} - \varvec{f}\right) + \Lambda _i, \end{aligned}$$

where here $\varvec{\mathscr {T}}$ and $\varvec{\mathscr {T}^{-1}}$ are the moments transform matrix and its inverse, respectively. The diagonal matrix $\varvec{S}$ indicates the relaxation rate of different moments and $\Lambda _i$ is a source term restoring Galilean-invariance to the bulk viscosity ^46,47^.

### Numerical simulation

Simulations have been carried out using our in-house LBM solver ALBORZ, which has already been thoroughly validated using a variety of benchmark studies ^46,48,49^. To make sure simulations are converging to the incompressible limit and to optimize computational costs, we conducted extensive testing of different Courant- Friedrichs-Lewy (CFL) numbers. We found that lower CFL numbers resulted in extensive simulation times, while higher CFL numbers led to pronounced compressibility effects. A specific CFL value of 0.052 was selected, which is optimal for achieving reliable results within a reasonable computational cost. This value ensures that the time-step size remains sufficiently small to accurately capture flow fluctuations without introducing numerical instabilities. By adhering to this CFL value, the extracted results represent converged physical data, rather than being influenced by numerical artifacts such as Gibbs oscillations.

Additionally, blood was assumed to be an incompressible fluid with a constant density and viscosity in Newtonian cases while the modified Cross model ^45^ was employed to depict the rheological properties of blood in the non-Newtonian scenario. Detailed numerical parameters for each case, including aneurysm status, cardiac periods, size, density ($\rho$) and viscosity ($\nu$) as well as core hours, are summarized in Table 1. It should be noted that the spatial ($\delta _r = 6.5 \times 10^{-5}$ m) and temporal ($\delta _r = 1.9 \times 10^{-6}$ s) resolutions are the same for all cases.

**Table 1:** Summary of parameters used in this study, encompassing aneurysm status, cardiac periods, size, density ($\rho$) and kinematic viscosity ($\nu$) as well as core hours.

Case ID	Status	Period [s]	Size [${\text{mm}}^{3}$]	$\rho\,[{\mathrm{g/cm}}^{3}]$]	${\nu\,[\text{Poise}]}$	Core-h
Patient A	Ruptured	1	21.20$\times 37.61 \times$19.70	1.000	0.040	8.76E3
Patient B	Unruptured	1	32.50$\times$18.40$\times$11.10	1.000	0.040	3.81E3
Patient C	Ruptured	0.81	12.29$\times$20.59$\times$14.06	1.055	0.038	2.06E3
Patient D	Unruptured	0.99	13.30$\times$14.56$\times$17.30	1.055	0.038	1.94E3
Patient E	Ruptured	0.99	59.26$\times$34.48$\times$34.98	1.000	0.040	3.90E4

**Figure 2:**
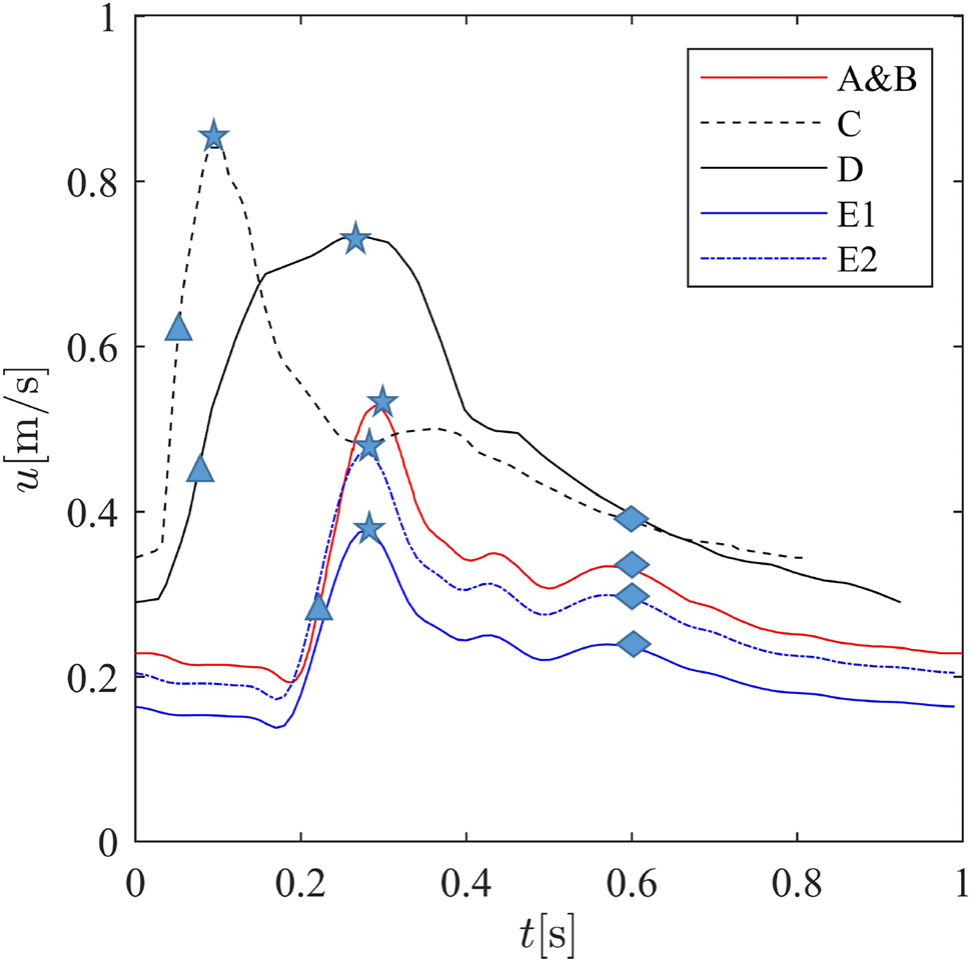
Illustration of the inflow velocity waveforms over an entire cardiac cycle for patients A &B (red) ^50^, C (black dashed) ^51^, D (black solid) ^51^ and E (blue) ^12^, respectively. The triangle, star and diamond present the acceleration (T1), peak (T2), and deceleration (T3) phases, respectively.

All simulations were conducted over three periods on the SuperMUC-NG supercomputer, utilizing 48-core Skylake nodes, with data sampling carried out during the third cycle in order to eliminate any potential effects stemming from initial transients. The wall-clock time for a single cardiac period ranged from 13 node-hours to 11 node-days involved 11 nodes.

**Boundary conditions:** All of the inlets and outlets were extended along the normal vector of the corresponding surfaces to reduce the possible effects of boundary conditions. Additionally, in the context of LBM, we employed curved boundary condition treatements to better represent the complex geometry of the vessel. This involves interpolating boundary conditions to align with the lattice structure, which helps minimize numerical artifacts and improve simulation accuracy near the boundaries. This approach is particularly beneficial for accurately capturing the flow dynamics within aneurysms, where the geometry can significantly impact the simulation results. For further details on the interpolated bounce-back scheme we refer readers to^52^. The inlet boundary conditions were configured with pulsatile velocity profiles depicted in Figure 2. It should be noted that case E was modeled using two different inflow curves (E1, E2) ^12^. The duration of each cardiac cycle (T) is shown in Table 1. Constant pressure was prescribed at outlets, primarily due to the limited availability of pressure measurements at the outlet or the lack of information regarding the correlation between flow rate and pressure. The walls were assumed to be rigid and no-slip boundary conditions were applied. The curved boundary condition was used to better represent the complex geometry of the aneurysms.

### Flow quantification

We employ two primary metrics to quantify and analyze the flow dynamics within intracranial aneurysms: Fluctuating (or turbulent) kinetic energy (FKE), power spectral density (PSD). Although wall shear stress is a critical parameter in understanding aneurysm hemodynamics, it is not calculated in this study due to limitations in our current solver. Specifically, the challenge lies in accurately determining the normal vector of the curved boundary wall at each node. We are actively working on developing this capability for future studies.

**Fluctuating kinetic energy**: FKE is a crucial metric for quantifying flow fluctuations ^26,33^. In this study, we will focus the majority of our analysis on fluctuating components of the velocity field. To quantify the variations, the instantaneous velocity is decomposed into a mean component $\overline{\varvec{u}}(\varvec{r},t)$ and a fluctuating component $\varvec{u}'(\varvec{r},t)$ where:

3 $$\begin{aligned} \overline{\varvec{u}}(\varvec{r},t) = \frac{1}{\Delta t}\int _{t_0}^{t_0+\Delta t} \varvec{u}(\varvec{r},t) dt, \end{aligned}$$

where $\Delta t$ is the averaging period and:

4 $$\begin{aligned} \varvec{u}'(\varvec{r},t) = \varvec{u}(\varvec{r},t) - \overline{\varvec{u}}(\varvec{r},t). \end{aligned}$$

FKE is expressed as:

5 $$\begin{aligned} \text{FKE}(\varvec{r},t) = \frac{1}{2\Delta t}\int _{t_0}^{t_0+\Delta t} \varvec{u}'(\varvec{r},t)\varvec{u}'(\varvec{r},t) dt. \end{aligned}$$

**Power spectral density:** PSD shows how the power of a signal is distributed over the frequency domain; this provides a quantitative measure of flow fluctuations ^24^. Here, the PSD at each probe point has been computed using Matlab (MathWorks inc.) based on Welch’s method.

The discrete-time power spectral density ($S_{xx}(f)$) is defined at frequency *f* by ^53^:

6 $$\begin{aligned} S_{xx}(f) = \frac{\delta _t^2}{\Delta t}\left| \sum _{t=t_0}^{t_0+\Delta t}\phi (t) \exp {\left[ -2\pi \sqrt{-1}f (t-t_0)\right] } \right| ^2 \end{aligned}$$

where $\delta t$ and $\Delta t$ are the sample interval and the length of the signal, respectively, while $\phi (t)$ is the velocity signal.

## Results

The following section presents qualitative and quantitative comparisons of hemodynamic simulations among the cases, considering both Newtonian and non-Newtonian models. The grid-independent analysis can be found in the supplementary information S. 2.1.

### Ruptured vs. unruptured aneurysms

**Flow patterns within the aneurysm sac–**

**Figure 3:**
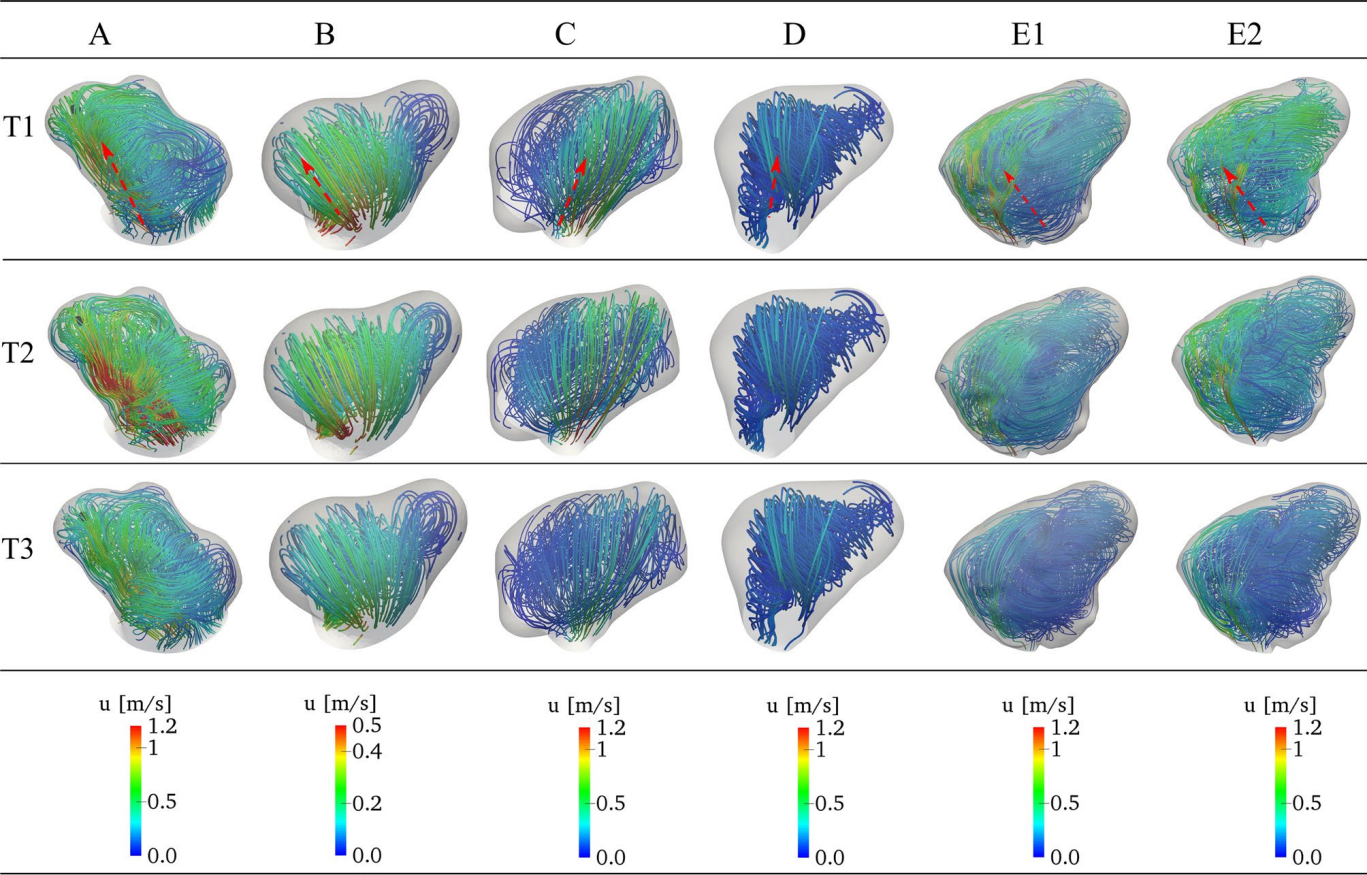
Flow streamlines colored by flow velocity inside the aneurysm sac for all cases at the acceleration (T1), peak (T2), and deceleration (T3) systole under Newtonian assumption. The red arrow indicates a high-speed jet.

Figure 3 provides a qualitative comparison of flow streamlines within the aneurysm sac under the Newtonian scenario, offering insights into the dynamic behavior during the acceleration, peak, and deceleration phases of the cardiac cycle (see Figure 2). The initial comparison reveals distinctions between flow streamlines in ruptured and unruptured cases. For the ruptured aneurysms (A, C, E), a concentrated high-speed jet originating from the parent vessel, striking the opposing wall at a specific angle, generates intricate vortical structures and disturbed flow patterns. In contrast, the unruptured cases (B, D) present a quite stable flow pattern characterized by a single large-scale vortex that persists consistently over all three periods. Generally, the flow streamlines display a relatively smoother and more laminar flow in unruptured cases. Additionally, for case E, since it is from Steinman’s CFD Challenge ^12^, we also compare our results to those from other solvers. A detailed comparison can be found in the supplementary document.

This first, qualitative analysis revealed a stronger intrasaccular flow with larger variations in time in the ruptured cases. Therefore, velocity fluctuations in time appear to play an important role and will be quantified in what follows.

**Figure 4:**
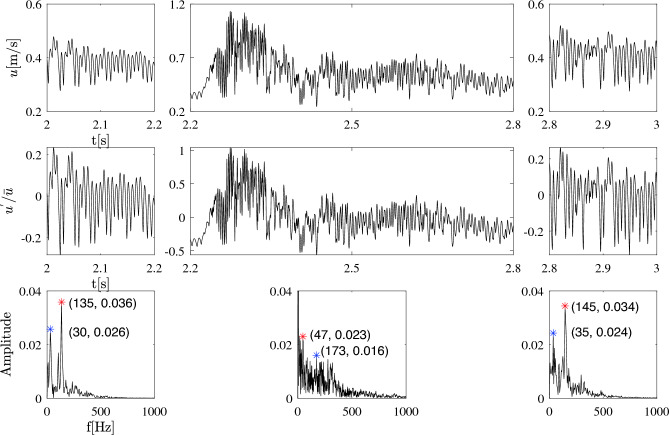
Time history of velocity, normalized fluctuating velocity, and fast Fourier transform (FFT) of velocity at the rupture point P6 in case A in the Newtonian scenario. The red and blue stars in the bottom row indicate the first and second dominant frequencies.

**Temporal velocity fluctuation in the rupture point–** The profiles of velocity over time in all monitoring points are shown and the findings are further examined in the supplementary document S. 2.2. To better study the velocity fluctuations at the known rupture point (P6) in case A, one cardiac cycle was divided into three distinct phases (2-2.2 s, 2.2-2.8 s, 2.8-3.0 s) (see Figure 4), facilitating clearer analysis. Examining the top row of the plot, at the commencement of the cardiac cycle (left subfigure), fluctuations appear uniform and the average velocity hovers around 0.4 m/s. Subsequently, substantial fluctuations manifest after peak systole, driving the maximum velocity to almost 1.2 m/s. In the final phase, the flow instability diminishes toward the end of the deceleration phase, transitioning into low-frequency fluctuations with an average velocity of approximately 0.4 m/s (right figure), closely resembling the flow characteristics observed at the start of the cardiac cycle (compare left and right subfigures).

The middle row exhibits the normalized fluctuating velocity profile over time. It reveals that the relative amplitude of the velocity fluctuations is roughly 20% during the early and late phases of the cardiac cycle, increasing significantly to around 100% during peak systole.

**Figure 5:**
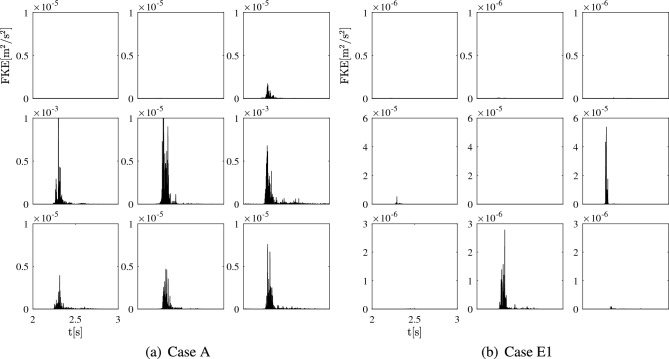
Time evolution of FKE in the cases A and E1 at all monitoring points, considering blood as a Newtonian fluid. From top left to bottom right, results at points P1 to P9 are shown. Note the different vertical scales.

The bottom row depicts the fast Fourier transform (FFT) of the fluctuating velocity signal. This analysis indicates that the dominant frequencies (represented by red stars) are approximately 140 Hz during both the early and late phases of the cardiac cycle. During peak systole, the frequency spectrum becomes more complex, with multiple frequencies participating and the FFT signal becoming highly populated, indicative of the onset of turbulent conditions. In this phase, the dominant frequency is roughly 47 Hz, accompanied by a secondary peak at 173 Hz.

The lower-frequency fluctuation observed here may possibly be attributed to cycle-to-cycle fluctuations arising as the flow decelerates, a phenomenon previously documented in studies ^33,54^.

**Fluctuating kinetic energy (FKE)–** A more in-depth analysis of cases A and E is conducted by calculating FKE. As demonstrated in Figure 5, the distribution of FKE at the monitoring points in rupture cases (A and E1) offers valuable insights. Notably, the time evolution of FKE within the parent artery (points P1 &P2) remains consistently at zero for both cases. This absence of FKE aligns with the smooth velocity profiles and absence of discernible fluctuations in these regions. Furthermore, the peaks of maximum FKE primarily occur during peak systole in both cases. However, uniquely in case A, distinct fluctuations are also observed at the rupture site (P6) during late systole, with the second-highest peak reaching $\sim 1\times 10^{-4}~\mathrm {m^2/s^2}$. This value surpasses the overall peak FKE at all other points both within and outside the aneurysm sac. Interestingly, the highest level of FKE also occurs at P6 in case E1 (orders of magnitude larger, $\sim 6\times 10^{-5}~\mathrm {m^2/s^2}$).

At the bifurcation point P3 of case A, a low level of FKE ($\sim 2\times 10^{-6}~\mathrm {m^2/s^2}$) emerges during peak systole, revealing the initiation of flow instability. In contrast, FKE at this bifurcation in case E1 remains at negligible levels. At the outlets, remarkably pronounced fluctuations are also apparent, with a noteworthy disparity in FKE between both outlets in case A. This variance can potentially be attributed to the specific geometry of the ruptured aneurysm and vessel tree configuration. It is noteworthy that the flow rate exiting through the outlet associated with P4 greatly surpasses that of the outlet containing P5. Minor fluctuations are also discernible at the outlet of case E, with FKE at approximately $\sim 5\times 10^{-5}~\mathrm {m^2/s^2}$ while the FKE level is zero at the other outlet (P5).

The remaining points within the aneurysm sac of case A (P7, P8, P9) exhibit lower FKE values compared to the other monitoring points. At the start and conclusion of the cardiac cycle, FKE effectively diminishes to near zero in these areas. Likewise, FKE values at points (P5, P7, P9) in case E1 also converge to zero. However, at point P8, the FKE level remains around $\sim 3\times 10^{-6}~\mathrm {m^2/s^2}$.

Overall, these distinctive features of FKE suggest that intensive flow instability, as measured by FKE, may serve as a characteristic hallmark of the potential rupture location within the aneurysm sac.

**Figure 6:**
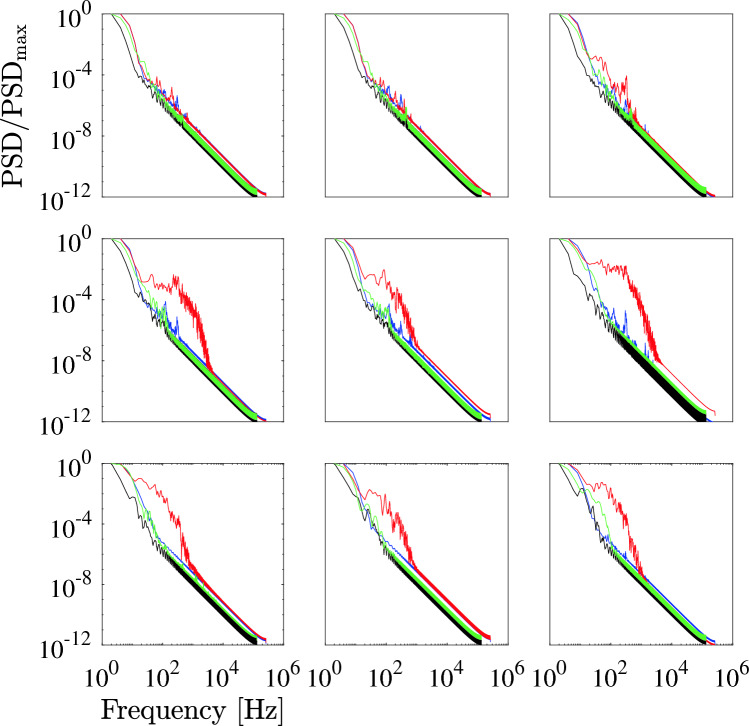
Energy spectra of velocity in case A (red), case B (blue), case C (green) and case D (black) at the nine monitoring points in the Newtonian case. From top left to bottom right, results at points P1 to P9 are shown.

**Power spectral density–** Figure 6 shows the energy spectra of velocity at the probes in cases (A, B, C, and D). Notably, within the aneurysm sac, the PSD of case A consistently ranks the highest in comparison to the other cases. Case A shows distinctive spectral characteristics indicative of turbulent-like flow with pronounced fluctuations. A notable and expansive rise in PSD is discernible around the approximate frequency of 100 Hz. This unique behavior is consistent across all points from P4 to P9 within the aneurysm sac. Conversely, the PSD of the flow velocity for cases B, C, and D reveal a nearly monotonic decline relative to frequency, devoid of prominent peaks. Overall, the simulation outcomes unequivocally affirm that the flow in case A exhibits distinct spectral traits divergent from the other cases, particularly conspicuous at the rupture point P6. At this specific location, a clear frequency peak emerges at approximately 120 Hz prior to peak systole, hinting at the potential significance of high-frequency features in the context of aneurysm rupture.

**Figure 7:**
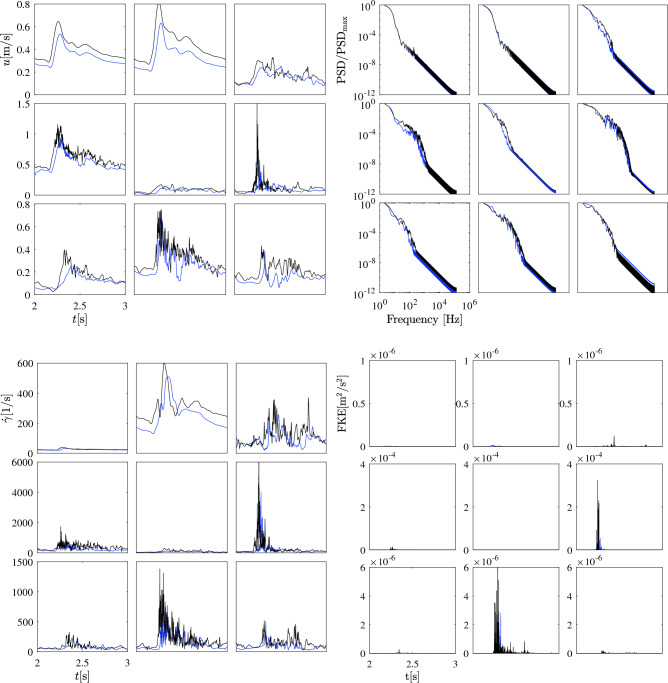
Illustration of velocity magnitude, PSD, shear rate, FKE in case E with flow rates E1 (blue) and E2 (black) at the nine monitoring points. From top left to bottom right, results at points P1 to P9 are shown in a row-wise order. Note the different vertical scales.

**Impact of flow rate on aneurysm dynamics–**

Figure 7 illustrates the evolution of velocity magnitude, PSD over frequency, shear rate over time, and the FKE over time for case E under two distinct inlet flow rates (E1 & E2). This figure shows that an increase in the inlet velocity profile leads to enhanced velocity fluctuations in both intensity and frequency. At point P6, the disparity can be as substantial as threefold. While the PSD profile shows relatively minimal divergence, noticeable fluctuations emerge in the shear rate profile. These fluctuations are evident not only at point P6 but also at the other points within the entire sac. The FKE profile presents the maximal velocity fluctuation at P6 and P8, attaining values of $4\times 10^{-4}$ and $6\times 10^{-6}$ $\mathrm {m^2/s^2}$, respectively.

### Comparison of Newtonian and non-Newtonian models

**Figure 8:**
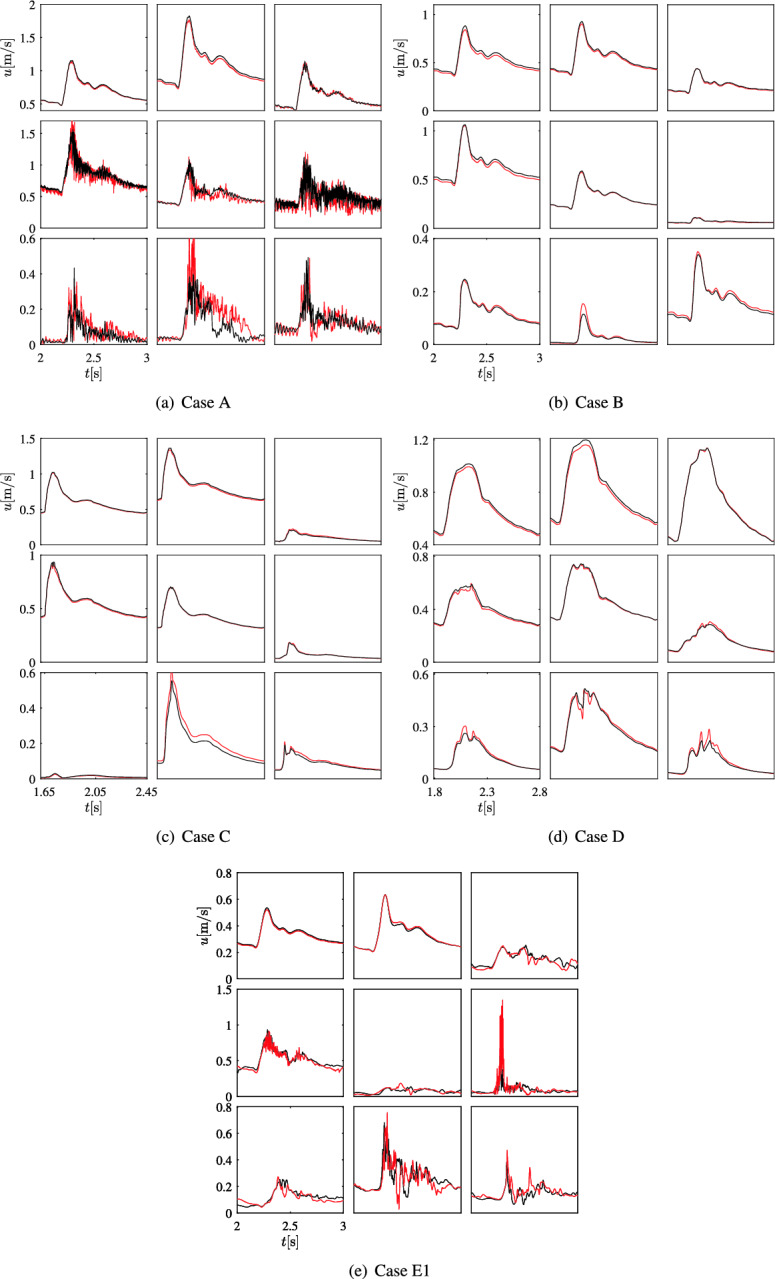
Velocity over time in all cases at the nine monitoring points. The black and red solid lines denote the velocity magnitude computed using a Newtonian and a non-Newtonian (Cross) model, respectively. From top left to bottom right, results at points P1 to P9 are shown in a row-wise order. Note the different vertical scales.

Figure 8 presents the velocity fluctuations between ruptured and unruptured cases. In cases B, C, and D, the non-Newtonian model displays minimal impact, as indicated by nearly overlapping black and red curves. Notably, in case A, discernible differences emerge, particularly at point P8 within the sac. High-frequency fluctuations are absent in case B, regardless of the fluid behavior assumption. The situation contrasts significantly in case A where the impact of the non-Newtonian model is negligible in the parent artery (points P1& P2) and at the neck (P3) while the velocity fluctuations are much stronger when considering a non-Newtonian behavior, particularly at point P6 and point P8. Similar tendencies are evident in case E1, where the non-Newtonian model substantially impacts points P6 and P8. The energy spectra of the velocity and shear rate are computed in all cases in the supplementary material S. 2.5.

## Discussion

This study provides insights into the hemodynamic characteristics of ruptured and unruptured, patient-specific intracranial aneurysms using high-resolution LBM simulations. Our study underscores the significance of qualitative hemodynamic parameters in predicting the rupture status of intracranial aneurysms. We observed that complex flow patterns, inflow concentration, unstable flow, and a smaller flow impingement zone played pivotal roles in assessing the rupture risk of aneurysms. These qualitative findings corroborate prior research indicating that ruptured aneurysms tend to exhibit flow patterns characterized by changing direction of the blood inflow jet, resulting in the formation of a single vortex ^27^. The inclusion of such qualitative hemodynamic parameters enriches the predictive capability for intracranial aneurysm rupture status.

In addition to qualitative metrics, the grid-independent analysis confirms the presence of high-frequency fluctuations within ruptured intracranial aneurysms, aligning qualitatively with earlier numerical investigations ^55–57^. However, these high-frequency fluctuations are not consistent across all rupture cases, as evidenced by case C. This variability underscores the complex nature of aneurysm hemodynamics and the need for further research to elucidate the underlying mechanisms governing rupture. The high spatial and temporal resolution used in this study is essential for investigating these high-frequency fluctuations, particularly in cases A and E. Using a coarser grid and a large time step can result in increased numerical dissipation, artificially damping the onset of flow instability. Our findings suggest that elevated inlet velocity may magnify fluctuations, potentially contributing to an augmented risk of aneurysm rupture. While it is expected that flow instabilities increase with flow rate due to the higher Reynolds number, the specific implications in the context of rupture susceptibility require further investigation.

Our investigations also highlight the significant impact of non-Newtonian models on shear rate at specific locations, particularly around peak systole, which is more pronounced in ruptured aneurysms. Interestingly, the exact rupture point is almost unaffected by the shear rate, indicating the need for further studies involving additional configurations to clarify these observations. Given that shear rates may not always exceed 100 ${\text{s}^{-1}}$, employing a suitable non-Newtonian model is recommended, especially when assessing flow instability in patient-specific aneurysm. There are still disagreements within the scientific literature concerning the significance of non-Newtonian features, particularly regarding intracranial aneurysms. Although some studies claim that there is no significant impact of non-Newtonian models ^58,59^, others concluded that non-Newtonian behavior can affect the numerical results ^60,61^. Recent investigations by Hosseini et al. ^45^ have delved into the impact of non-Newtonian behavior on the flow distribution within aneurysms. Their findings indicate that the adoption of a non-Newtonian model significantly influences the flow field, particularly within the aneurysm sac and in proximity to its walls, given the prevalence of low shear rates across substantial portions of the sac. Streamlines have been qualitatively compared between Newtonian and non-Newtonian cases among these aneurysms, revealing evident distinctions in the ruptured cases. In the analysis of energy spectra and shear rate, it is concluded that the non-Newtonian model tends to induce higher amplitudes of shear rate fluctuations, with notable differences observed at specific points. Notably, the rupture point P6 in case A, which is somewhat unexpected based on the analysis of energy spectra and shear rate across all cases. Further comparison detailed analysis can be found in the supplementary information S. 2.5.

While the complex interplay between hemodynamic factors and biological processes regarding aneurysm formation and rupture remain poorly understood, recent progress has been made in understanding additional risk factors and mechanisms underlying aneurysm pathophysiology. For instance, Wang et al. proposed a potential self-amplification mechanism of tissue degradation, driven by an inverse correlation between time-averaged wall shear stress and oscillatory shear index ^62^. Additionally, variations of wall shear stress (WSS) in the vicinity of flow-impingement regions have been investigated, shedding light on their potential significance in aneurysm development and rupture ^63^.

Several limitations should be mentioned in connection with the present study. Firstly, a limited number of configurations is considered. It is well known that aneurysm size, shape, location, the ratio of size and neck will affect hemodynamics and aneurysm rupture. A much larger number of patient-specific configurations must be considered in future studies to obtain statistically significant results. Secondly, all vessels have been assumed to be rigid. Clearly, the obtained findings might be modified when taking into account fluid-structure interactions; however, this would only be possible when having a detailed knowledge of wall resistance and structure, which is extremely challenging ^64,65^. Additionally, this study does not consider WSS and its fluctuations, which are crucial for understanding the mechanobiological factors leading to aneurysm rupture. Future work should include WSS analysis to provide a more comprehensive understanding of the hemodynamic stresses acting on the aneurysm walls. Nonetheless, it is important to acknowledge that uncertainties remain regarding the existence of fluctuations when the number of monitored points is increased. Further investigation would be required to ascertain whether fluctuations persist in the presence of additional monitoring points across the entire sac.

In conclusion, this study contributes to the evolving understanding of intracranial aneurysm hemodynamics, emphasizing the critical role of high-resolution simulations and the influence of non-Newtonian behavior on flow dynamics. Furthermore, best-practice rules are needed for the assessment of aneurysm hemodynamics using numerical simulations, in order to support more robust and reliable comparisons among studies ^39,66,67^. Ultimately, this would open the door for an efficient support of clinicians, leading to personalized treatment options.

## Conclusion

This study provides insights into the hemodynamic characteristics of ruptured and unruptured, patient-specific intracranial aneurysms. High-frequency fluctuations within ruptured aneurysms were identified, highlighting the need for high-resolution simulations. Our findings suggest that elevated inlet velocity may magnify fluctuations, potentially contributing to an augmented risk of aneurysm rupture. Further research with a broader range of patient-specific cases is needed to fully elucidate the relationships between non-Newtonian effects, flow dynamics, and rupture risk in patient-specific intracranial aneurysms.

### Supplementary Information

Supplementary Information.

## Data Availability

The datasets used and/or analysed during the current study available from the corresponding author on reasonable request.
