# Supplementary material for: On flow fluctuations in ruptured and unruptured intracranial aneurysms: resolved numerical study
Source: Sci Rep. 2024 Aug 23;14:19658. doi: 10.1038/s41598-024-70340-7 (PMC11344026; doi:10.1038/s41598-024-70340-7)
Supplement: Supplementary file 1 — Supplementary Information. [file 41598_2024_70340_MOESM1_ESM.pdf]

# 1 Supplementary Notes

## 1.1 Imaging and Segmentation

**Patient A** This aneurysm was detected in the posterior inferior cerebellar artery (PICA) in a 51-year-old female patient. The rupture location is known within the aneurysm sac. The 3D-DSA scan was carried out on an AXIOM-Artis (Siemens Healthineers AG, Forchheim, Germany) with the same spatial resolution (0.28 mm) as in the other case. The raw images were reconstructed using the 'EE/auto' kernel (Siemens Healthcare GmbH, Forschheim, Germany). The geometric models of the aneurysms were reconstructed on this basis and further processed to simplify numerical simulations, retaining only the intracranial aneurysms and the corresponding major arteries to guarantee reasonable inflow and outflow, while the distal arterial segments were cut off.

**Patient B** The unruptured aneurysm (of size 1.6 mm) was found in the middle cerebral artery (MCA) in a 53-year-old female patient who experienced an acute subarachnoid hemorrhage and severe headaches and was treated by coiling. The three-dimensional rotational angiography (3DRA) was performed on an Artis Q angiography system (Siemens Healthineers AG, Forchheim, Germany) with a spatial resolution of $0.28 \times 0.28 \times 0.28$ mm to produce tomography slices of the volume of interest. Subsequently, the reconstructed raw image was acquired from a syngo X Workplace (Siemens Healthcare GmbH, Forchheim, Germany) through an 'HU auto' kernel. Further details can be found in reference[1].

**Patient C and D** The two patient-specific aneurysms were roughly matched based on sex (female), age (83 years old for patient C and 63 years for patient D), location (middle cerebral artery), size (11.7 mm maximum diameter for patient C and 9.6 mm for patient D), and similar shape. Additional information about the patient's medical histories can be found in the reference[2,3].

**Patient E** In order to study the effect of Reynolds number on the flow instability, the ruptured giant cerebral aneurysm(of size 26 mm) from Steinman et al.[4] was selected, which was detected in the left internal carotid artery (ICA) in a 62 years old female patient. The nominal inlet diameter was measured to be 5.58 mm, and the corresponding peak Reynolds numbers were 513 and 651, respectively. Further information regarding the patients' histories is available in reference[5].

# 2 Supplementary Discussion

## 2.1 Impact of grid resolution on flow characteristics

In order to obtain accurate results while keeping acceptable computational costs, we first conducted a grid-independence study on case A and B considering Newtonian simulations. The grid size and time-step were varied simultaneously (keeping Courant-Friedrichs-Lewy (CFL) number constant) from 260 $\mu$m down to 65 $\mu$m and from 7.6 $\mu$s to 1.9 $\mu$s, respectively.

As expected, the high-frequency fluctuations were found to be remarkably sensitive to both temporal and spatial resolutions. As shown in the top row of Figure S1, the low-resolution simulation (Low resolution (LR)) failed to capture any high-frequency fluctuation across the entire domain for both cases. Furthermore, it exhibited pronounced discrepancies when compared to velocity profiles acquired at higher resolutions for both aneurysms. Specifically, in both intermediate (Middle resolution (MR)) and high-resolution (High resolution (HR)) simulations, the noticeable high-frequency oscillations were evident under the exact conditions and at the same locations, specifically at points P3 to P9 within case A. However, these fluctuations are entirely absent in case B.

Still, minor discrepancies are still visible between MR and HR for case A at points P7 and P8, within the aneurysm sac. These discrepancies could be attributed partly to the fact that the monitoring points were fixed to the nearest grid points, causing a slight shift in actual position between resolutions. It is also possible that the resolution HR still has not reached complete grid-independence. However, it must be kept in mind that the central objective of this study is to detect high-frequency fluctuations in cases where they appear. In that regard, the outcomes derived from MR and HR simulations are fully identical, revealing such fluctuations at identical positions for the equivalent conditions and with similar spectra. Taking into account that the total computational load is high but still acceptable using High-Performance Computers for the highest resolution (HR), it was decided to maintain this resolution for the subsequent sections of this paper to optimize precision.

On the other hand, a similar conclusion can be drawn from the spectral analysis. The spectral profiles derived from both MR and HR simulations are nearly indistinguishable, as depicted in the bottom row of Figure S1. This alignment indicates a remarkably consistent flow behavior between the two. In contrast, the spectra from the lower resolution LR simulations reveal a reduced energy content in comparison to the higher resolution outcomes in patient A, signifying a relatively laminar flow pattern. It is worth noting that the energy level remains consistently lower across all three simulations in patient B, serving as evidence of a laminar flow configuration.

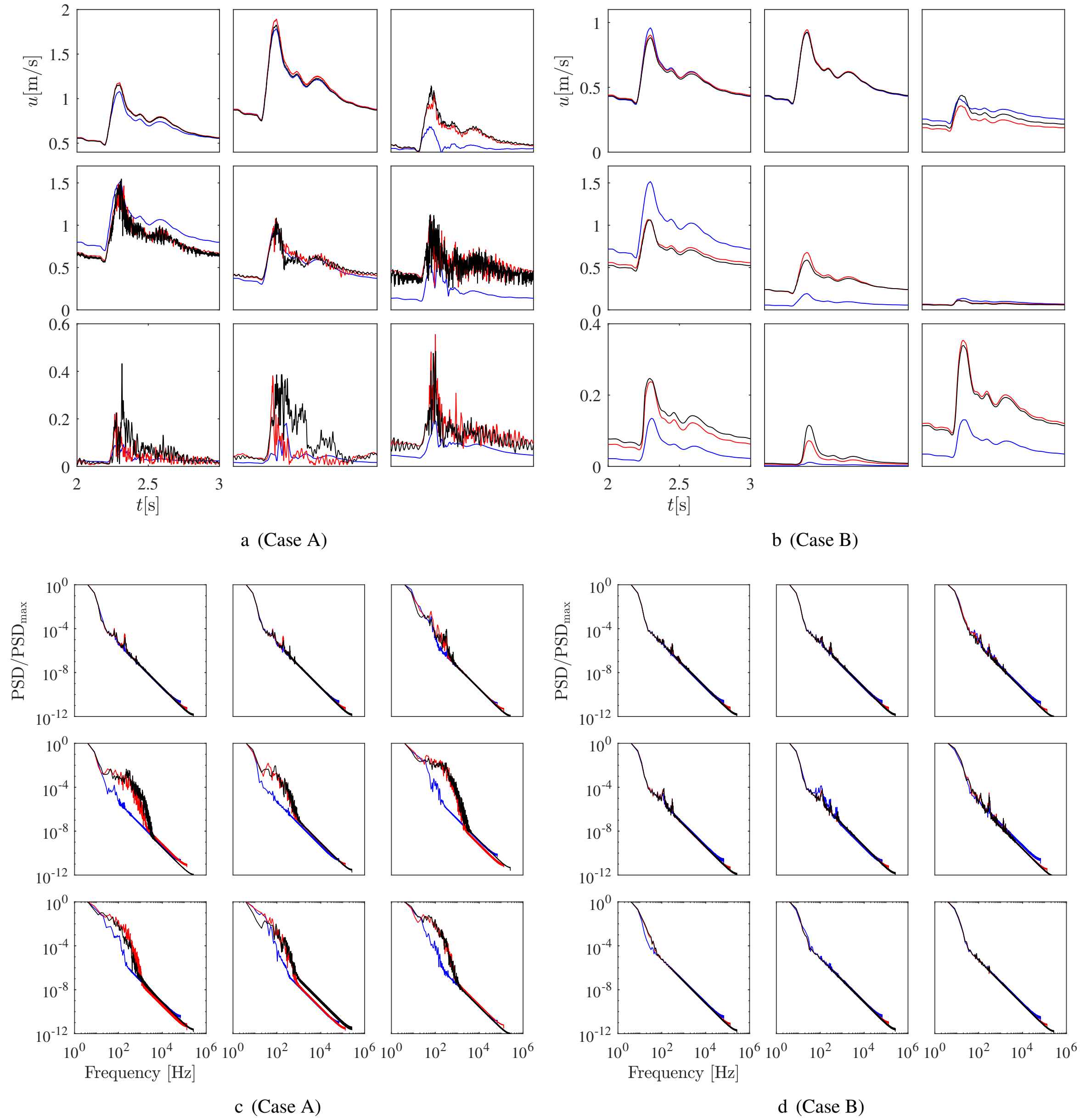


**Figure S1.** Time histories of the simulated velocity (Top row) and corresponding energy spectra of the velocity distributions(Bottom row) for patients A and B at 9 monitoring points. The blue, red and black solid lines denote the flow velocity and energy spectra for increasing resolution (LR: low, MR: middle, and HR: high, respectively). From top left to bottom right in a row-wise order, results at points P1 to P9 are shown.

Therefore, the choice of an appropriate resolution significantly influences the acquisition of accurate data concerning high-frequency oscillations. Opting for a lower resolution might potentially lead to an underestimation of the impact of high-frequency fluctuations in clinical assessments.

## 2.2 Temporal velocity fluctuation

To gain a more precise understanding of the occurrence and spatial distribution of flow fluctuations within the aneurysm sac, the findings should be further examined in the previous figure. Figure S1a shows the temporal variations of velocity, in particular at probe P6 in case A (remember that point P6 corresponds to the known rupture location in this case).

Firstly, it is important to note that discernible flow instabilities are absent in the parent artery (points P1&P2). Instead, flow fluctuations emerge initially at the bifurcation point (P3), and subsequently propagate throughout the entire sac and extend to the two outlets (points P4&P5). At bifurcation point P3, only subtle fluctuations are evident, primarily around the peak systole

phase. Similar dynamics have been reported by Le et al.[6], where a highly dynamic inflow jet was observed in the ostium of intracranial aneurysms. Their findings, through dynamic mode decomposition analysis, highlighted that high-frequency modes, characterized by brief duration, corresponded to flow separation at the proximal neck and the impingement of the jet onto the aneurysm wall. Within the aneurysm sac (points P6 to P9), both the frequency and amplitude of flow fluctuations exhibit notable escalation (as evident in the second row of Figure S1). On the other hand, as the flow passes beyond peak systole, both the amplitude and frequency gradually decay, even if velocity instabilities persist over the entire cardiac cycle in part of the sac. Interestingly, these fluctuations are particularly pronounced during the deceleration phase of the cardiac cycle. This phenomenon can be attributed to the suppression of transition effects during the acceleration phase and their enhancement during the deceleration phase, as also observed in pulsating flows in straight tubes[7].

Moving on to cases C and D in Figure S2b, a closer examination of the velocity profiles reveals a notable trend. In the nine monitored points within the sac, only minimal fluctuations can be observed. These fluctuations are nearly negligible, strongly implying the prevalence of laminar flow patterns in these cases.

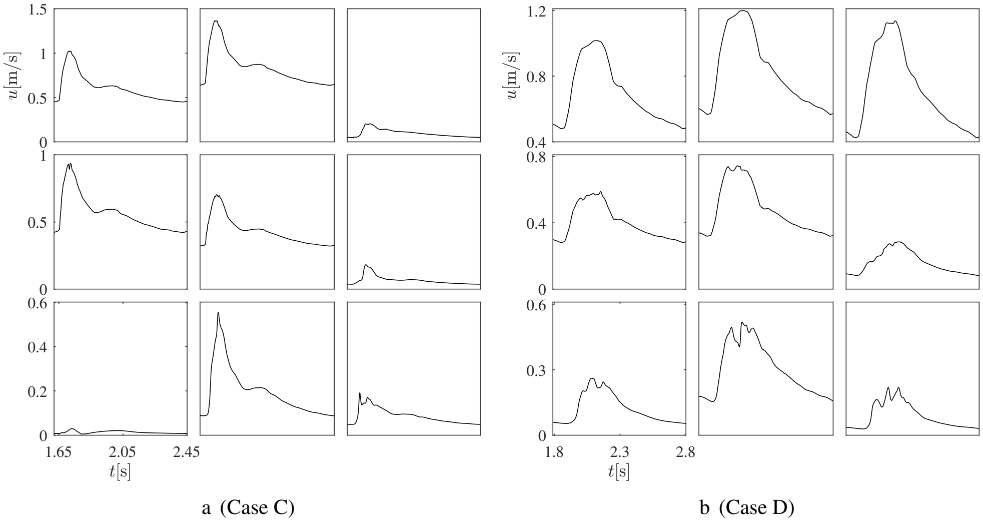


**Figure S2.** Time histories of the simulated velocity in cases C and D at nine monitoring points. From top left to bottom right in a row-wise order, results at points P1 to P9 are shown.

## 2.3 Power spectral density of shear rate

Figure S3 demonstrates the energy spectra of shear rate at the monitoring locations for the five cases. The PSD analysis reveals that the PSD values of both ruptured cases (A and E) are consistently higher compared to the other three cases.

Among ruptured cases, the case A exhibits a higher PSD than in case E. The higher PSD in case A suggests a greater magnitude of shear rate fluctuations, which could be indictive of hemodynamic disturbulences contributing to the rupture.

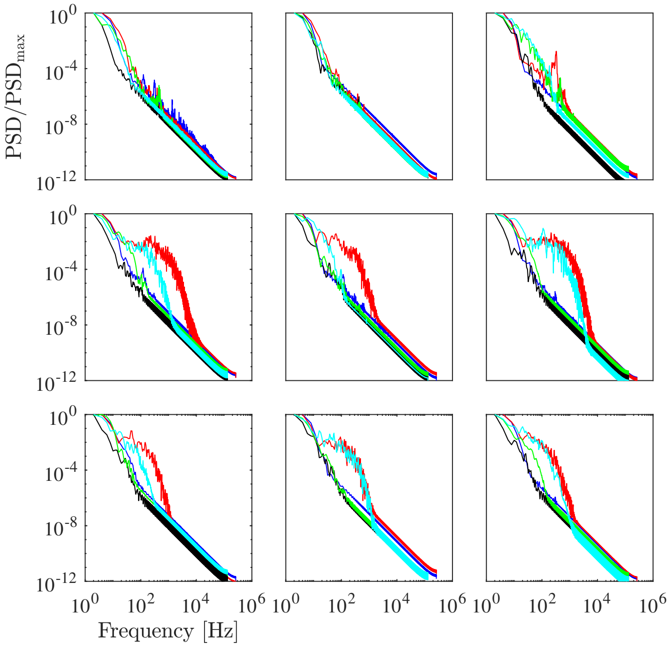


**Figure S3.** Energy spectra of shear rate in case A (red), case B (blue), case C (green), case D (black) and case E1 (cyan) at the nine monitoring points in the Newtonian case. From top left to bottom right in a row-wise order, results at points P1 to P9 are shown.

## 2.4 Comparison of Case E Results with CFD Challenge Solvers

Figure S4 shows the peak systolic velocities from 27 groups. Some solutions appear the evidence of flow fluctuations (U, W, and X) while our result and other groups (C, F, and G) reported smoother isosurfaces. Despite the smooth appearance, flow instabilities can still be observed at the monitoring points.

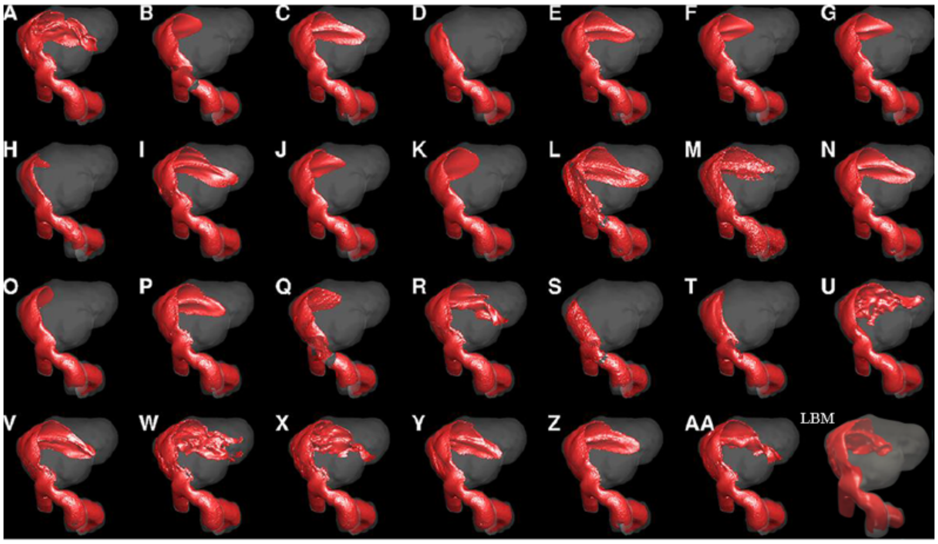


**Figure S4.** Shown are isosurfaces of velocity magnitude at 0.5 m/s at peak systolic: Current result (LBM) is shown at bottom right, others from Ref.[4]

## 2.5 Effect of non-Newtonian behavior

### *2.5.1 Flow pattern within the aneurysm sac*

Figure S5 provides a qualitative comparision of flow streamlines within the sac under the non-Newtonian model. One interesting observation is that while Newtonian and non-Newtonian streamlines are visually quite similar for the unruptured cases, they

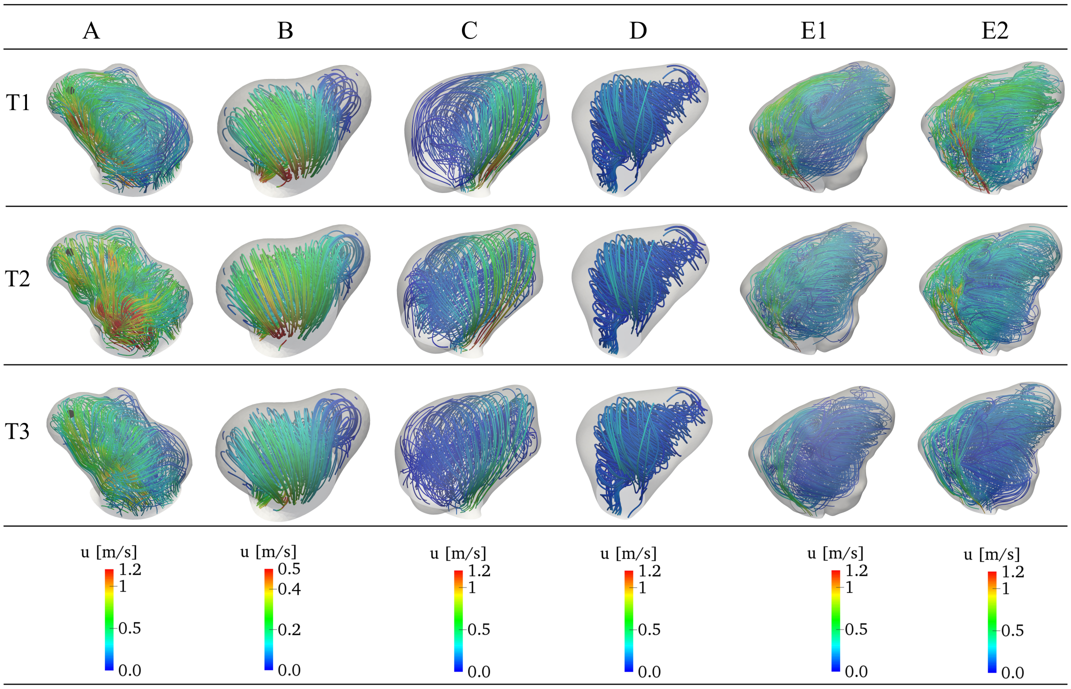


**Figure S5.** Flow streamlines colored by flow velocity inside the aneurysm sac for all cases at the acceleration (T1), peak (T2), and deceleration (T3) systole under non-Newtonian model.

exhibit obvious differences for the ruptured cases, also concerning large-scale flow structures.

#### *2.5.2 Quantitative analysis*

Figure S6 presents the energy spectra of the velocity in the ruptured cases (A&E1), unveiling higher energy levels in the non-Newtonian model compared to the Newtonian counterpart. These outcomes suggest that a non-Newtonian fluid behavior

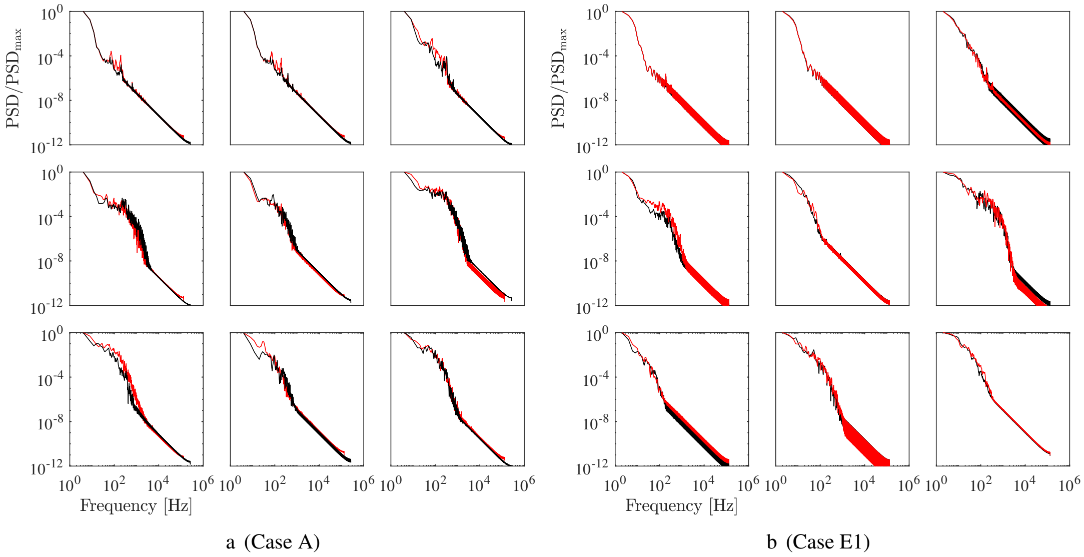


**Figure S6.** Energy spectra of the velocity over frequency in the case A and E1. The black and red solid lines denote the energy spectra computed using a Newtonian and a non-Newtonian (Cross) model, respectively. From top left to bottom right in a row-wise order, results at points P1 to P9 are shown.

may amplify flow instability. Additionally, the local shear rate at the various monitoring points among these cases is shown in Figure S7, investigating the potential impact of the different viscosity models. In the unruptured cases (B&D), the evolution of the shear rates exhibits similar trends under both Newtonian and non-Newtonian considerations, with peak systole-associated relative differences below 25%. Although high-frequency fluctuations are not observed in case B, there are weak velocity fluctuations within the sac in case D. Furthermore, the shear rates are quite small and stay below 1000 $s^{-1}$ at most of the points.

In the ruptured cases (A, C&E1), this correlation holds true primarily within the cerebral artery preceding the aneurysm(points P1&P2). The shear rate fluctuations remain modest at the bifurcation point (P3), yet considerable fluctuations are observable at points P4 to P9 in cases A and E1. The peak shear rate even exceeds 4000 $s^{-1}$ at point P6 and 1500 $s^{-1}$ at peak systole at the outlet P4 in both cases (that with the largest flow-rate, as discussed previously). This finding confirms the presence of significant velocity variations at these two points.

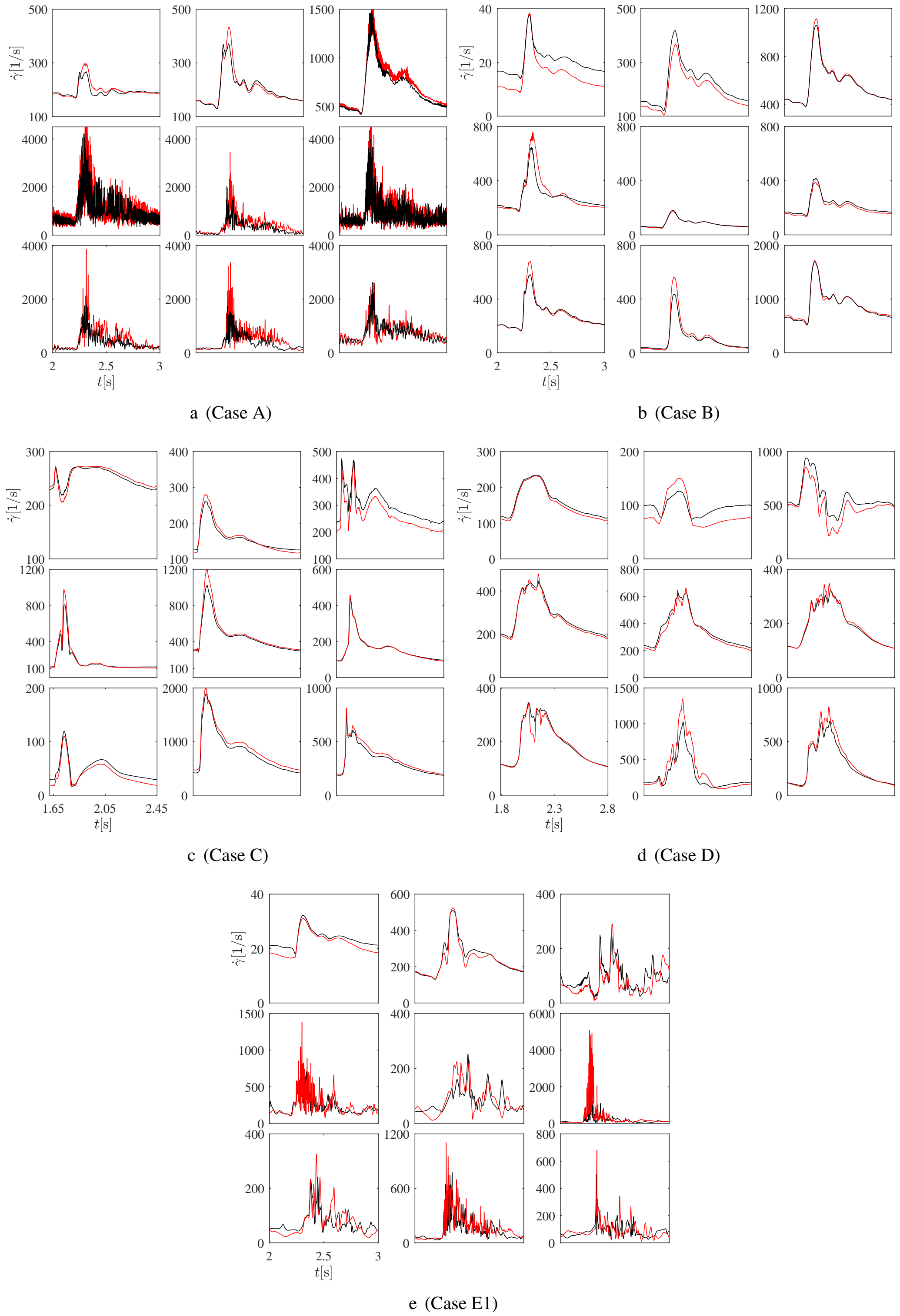


**Figure S7.** Shear rate over time in cases A, B, C, D and E1 at the nine monitoring points. The black and red solid lines denote the shear rate computed using a Newtonian or a non-Newtonian (Cross) model, respectively. From top left to bottom right in a row-wise order, results at points P1 to P9 are shown. Note the different vertical scales.

### 2.6 POD analysis

Figure S8 illustrates a combined qualitative illustration of the first two most energetic POD modes in ruptured (A) and unruptured (B) aneurysms as suggested by[8]. The temporal averaged flow information is represented by the first POD mode, therefore, it can effectively characterize the primary flow. It can be observed that the jet flow to the aneurysm sac has a higher velocity in the ruptured case A compared to the unruptured aneurysm B.

The second POD mode represents the secondary flow structures, which can be used to demonstrate the temporal variation of the blood flow. The higher POD modes are generally not straightforward to interpret physically. In Figure S8, the second mode is used to color-code the isosurface obtained from the first POD mode. The dark colors indicate stronger secondary flow features. The lighter colors associated with lower secondary flow features symbolize a stable flow throughout the cardiac cycle. This qualitative combined visualization of the first two POD modes enables the identification of high or low areas in the secondary flow and can efficiently help analyzing the complexity of the hemodynamics.

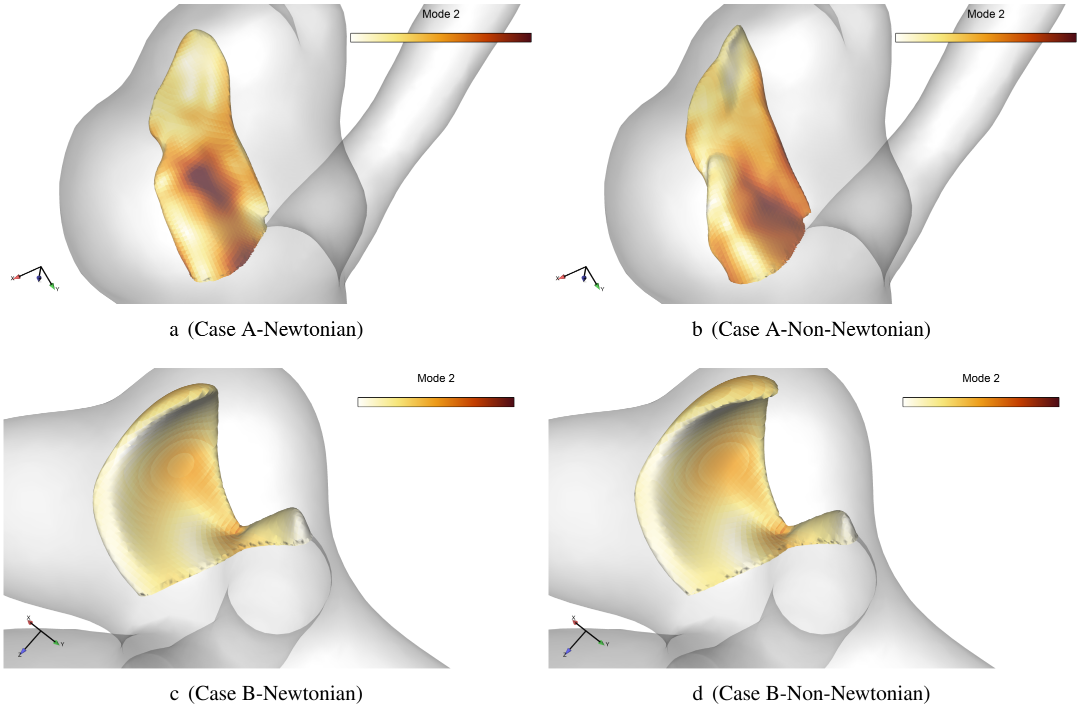


**Figure S8.** Hybrid visualization (based on[8]) of the isosurface obtained with the help of the first POD mode, but colored by the second POD mode for both aneurysms A (top, ruptured) and B (bottom, unruptured) with either Newtonian (left) or non-Newtonian models (right).

**Table S1.** Coordinates of the Probe Points in Five Cases

| Case \ Location | $P_1$ | $P_2$ | $P_3$ | $P_4$ | $P_5$ | $P_6$ | $P_7$ | $P_8$ | $P_9$ |
|---|---|---|---|---|---|---|---|---|---|
| A | (1.0117,1.0175,1.0110) | (1.0114,1.0097,1.0095) | (1.0103,1.0066,1.0071) | (1.0067,1.0064,1.0032) | (1.0128,1.0071,1.0102) | (1.0088,1.0009,1.0038) | (1.0074,1.0008,1.0070) | (1.0115,1.0019,1.0083) | (1.0081,1.0042,1.0072) |
| B | (1.9546,1.8281,1.8689) | (1.9622,1.8247,1.8662) | (1.9682,1.8213,1.8660) | (1.9719,1.8253,1.8650) | (1.9704,1.8161,1.8703) | (1.9697,1.8205,1.8642) | (1.9700,1.8186,1.8652) | (1.9701,1.8180,1.8668) | (1.9708,1.8203,1.8656) |
| C | (9.9980,10.0000,10.0000) | (10.0010,10.0010,9.9970) | (10.0025,10.0000,9.9945) | (10.0045,9.9960,9.9975) | (10.0040,10.0045,9.9945) | (10.0025,9.9950,9.9930) | (10.0010,10.0013,9.9909) | (10.0080,9.9980,9.9878) | (10.0090,9.9971,9.9915) |
| D | (0.9965,1.0080,1.0028) | (0.9983,1.0042,1.0010) | (0.9995,1.0010,0.9990) | (0.9980,0.9985,1.0060) | (1.0030,1.0055,0.9967) | (0.9975,0.9995,0.9999) | (0.9961,0.9950,0.9995) | (0.9990,0.9931,0.9961) | (1.0035,0.9930,1.0035) |
| E | (1.9900,2.0050,2.0030) | (2.0120,2.0130,2.0210) | (2.0160,2.0230,2.0210) | (2.0030,2.0165,2.0130) | (2.0200,2.0200,2.0195) | (2.0160,2.0300,2.0150) | (2.0245,2.0280,2.0140) | (2.0220,2.0240,2.0030) | (2.0300,2.0160,2.0030) |

## References


1. Voß, S., Beuing, O., Janiga, G. & Berg, P. Multiple aneurysms anatomy challenge 2018 (MATCH) -— Phase Ib: Effect of morphology on hemodynamics. *PLOS ONE* **14**, e0216813 (2019).
2. Janiga, G., Berg, P., Sugiyama, S., Kono, K. & Steinman, D. The Computational Fluid Dynamics Rupture Challenge 2013—Phase I: prediction of rupture status in intracranial aneurysms. *Am. J. Neuroradiol.* **36**, 530–536 (2015).
3. Berg, P. *et al.* The computational fluid dynamics rupture challenge 2013—phase II: variability of hemodynamic simulations in two intracranial aneurysms. *J. Biomech. Eng.* **137**, 121008 (2015).
4. Steinman, D. A. *et al.* Variability of computational fluid dynamics solutions for pressure and flow in a giant aneurysm: the ASME 2012 summer bioengineering conference CFD challenge. *J. Biomech. Eng.* **135** (2013).
5. Cebral, J. *et al.* Aneurysm rupture following treatment with flow-diverting stents: computational hemodynamics analysis of treatment. *Am. J. Neuroradiol.* **32**, 27–33 (2011).
6. Le, T. B. Dynamic modes of inflow jet in brain aneurysms. *J. Biomech.* **116**, 110238 (2021).
7. Juárez, L. H. & Ramos, E. Direct numerical simulation of transition to turbulence in an oscillatory channel flow. *Comptes Rendus Mecanique* **331**, 55–60 (2003).
8. Janiga, G. Novel feature-based visualization of the unsteady blood flow in intracranial aneurysms with the help of proper orthogonal decomposition (POD). *Comput. Med. Imaging Graph.* **73**, 30–38 (2019).
